# Activated Peritoneal Cavity B-1a Cells Possess Regulatory B Cell Properties

**DOI:** 10.1371/journal.pone.0088869

**Published:** 2014-02-13

**Authors:** Bram Margry, Saskia C. W. Kersemakers, Aad Hoek, Ger J. A. Arkesteijn, Willemien H. Wieland, Willem van Eden, Femke Broere

**Affiliations:** Department of Infectious Diseases and Immunology, Utrecht University, Utrecht, The Netherlands; Trinity College Dublin, Ireland

## Abstract

Previous studies have suggested that murine peritoneal cavity-derived B-1a cells possess similarities with described regulatory B cell subsets. The aim of the current study was to examine the potential immunoregulatory function of peritoneal cavity-derived B(-1a) cells. *In vitro* activation of peritoneal cavity-derived B- and B-1a cells shows that activation of these B cells with anti-CD40 and LPS induces these cells to secrete more IL-10, IL-6 and IgM as compared to splenic B cells. In a suppression assay, CD40/TLR4-activated peritoneal cavity B cells possess regulatory B cell functions as they inhibit the capacity of CD4^+^ T cells to produce both tumor necrosis factor-α and interferon-γ. Splenic B cells did not show this, whereas non-activated peritoneal cavity B cells augmented the capacity of CD4^+^ T cells to produce tumor necrosis factor-α, while the ability to produce interferon-γ was not altered. The current paper compares splenic B cells to peritoneal cavity B(-1a) cells in an *in vitro* activation- and an suppression-assay and concludes that peritoneal cavity B(-1a) cells possess properties that appear similar to splenic autoimmune-suppressive regulatory B cell subsets described in the literature.

## Introduction

Research in the past decade has convincingly shown that certain B cell subsets, nowadays referred to as “regulatory B cells” (Bregs), possess the capacity to down-regulate immune-responses via the secretion of interleukin (IL)-10. There is no definite surface marker or master-transcription factor to identify Bregs, and they are functionally defined by their immune-suppressive action, either *in vitro* or *in vivo* [1]–[3]. The best described Bregs, with well confirmed regulatory functions, include the splenic CD21^hi^CD23^hi^CD1d^hi^ transitional 2 marginal zone precursor B cells described by the group of Mauri [1], [4] and the IL-10-producing B cells termed ‘B10’ cells characterized by the group of Tedder. The latter are mainly found within the CD1d^hi^CD5^+^ splenic B cell subset [3], [5].

B-1a cells are innate-like B cells that have the ability to produce polyreactive natural antibodies, secrete large amounts of cytokines (among others IL-10), and are known for their expression of CD5 [6]-[8]. There are multiple similarities between B-1a cells and specific Breg subsets (particularly Bregs with the CD5^+^ CD1d^hi^ B10 phenotype), but it is still unclear how these subsets are related, or if they are possibly even the exact same cells [1], [3], [6].

Bregs need to be activated in order to exert their suppressive functions, and activation of Bregs presumably takes place *in vivo* in the context of inflammation. Although the exact mechanism is incompletely understood, both the groups of Mauri and Tedder have shown that *in vivo* activated Bregs are more potent suppressors of autoimmunity than their non-activated counterparts [4], [9]. There is evidence that this activation is antigen-specific, since Bregs that are *in vivo* activated by one antigen (Ag), do not protect in inflammatory models induced by another Ag [4], [5]. *In vitro*, Bregs can be activated in an antigen-nonspecific way to secrete IL-10 and suppress by a variety of stimuli that include activation through Toll-like receptor (TLR)-ligands [10]–[13], CD40 [12], [14], [15], a combination of these two [9], [12], [16], or the cytokine IL-21 [9].

Breg-mediated amelioration of autoimmune diseases in animal models (*e.g.* antigen-induced arthritis, collagen-induced arthritis and experimental autoimmune encephalomyelitis) is typically dependent upon IL-10 but besides that relatively little is known about the mechanism of action. Most reports indicate that Bregs influence T cell activation. Protection induced via the adoptive transfer of Bregs often correlates with a reduction in interferon (IFN)-γ-, IL-17- and/or tumor necrosis factor (TNF)-α-positive T cells [4], [9], [17], [18] and sometimes increased levels of Foxp3^+^ regulatory T cell (Tregs) [19] or IL-10-producing T cells [20]. Furthermore, B cell depleted mice or studies using IL10^−/−^ B cells show that B cell-derived IL-10 is needed *in vivo* to maintain the levels of IL-10-producing T cells [18],[21] and Foxp3-positive Tregs [18], [22] found in wild type mice.

*In vitro* Breg suppression assays are sometimes used to decipher immunosuppressive mechanisms. Although, Bregs are reported to limit T cell proliferation *in vivo* [4], [9] in a similar way as Tregs do, this is only occasionally seen in *in vitro* Breg suppression assays [23] and most reports do not detect this type of inhibition [10], [17]. Instead, Breg suppression assays show that Breg-derived IL-10 inhibits the promotion of proinflammatory cytokine (IFN-γ and TNF-α) positive CD4^+^ T cells [15], [17] the production of TNF-α by monocytes [11], [24] or T cell activation by dendritic cells [10], [17]. Human Bregs are reported to possess identical functions *in vitro* [25]–[27].

The capacity to produce IL-10 is a *sine qua non* for a Breg, but the capacity of a B-1 cell to produce this cytokine does not automatically define B-1 cells as Bregs. IL-10 is a pleiotropic cytokine with a variety of functions [28], and the exact function exerted may depend upon several micro environmental factors *e.g.* other cytokines secreted by the same B cell. Furthermore it has been demonstrated that B cells with IL-10-secreting capabilities often possess the ability to secrete IL-6 as well, and B-cell derived IL-6 plays a prominent role in the pathogenesis of autoimmune diseases [29]. Numerous other studies aimed to elucidate the exact phenotype of Bregs, and found that their phenotype partially overlaps with (splenic) B-1a cells [3], [5]. This signifies that populations deemed Bregs (and isolated as such) contain B-1a cells as well, either as an ‘irrelevant contaminant’ or possibly as the actually functional immunosuppressive cell. In the current paper, we examined whether the well-defined B-1a cell containing peritoneal cavity B cell population possessed an immunoregulatory function.

## Materials and Methods

### Mice and Ethic Statement

Female BALB/c mice (10–12 weeks old) were purchased from Charles River Laboratories (Maastricht, the Netherlands) and kept under standard housing conditions at the Central Animal Laboratory of the Utrecht University. All animal experiments were approved by the Animal Ethics Committee from the Utrecht University (DEC Numbers: 2011.II.05.90, 012.II.08.108 and 2012.II.11.157). All efforts were made to minimize animal discomfort.

### Cell isolation

Peritoneal cavity (PerC) washout cells were obtained by an i.p. injection of 2 ml air and 8 ml of phosphate buffered saline supplemented with 2% fetal calf serum and 2 mM ethylenediaminetetraacetic acid (Gibco) that was subsequently collected using a transfer pipette. The washout cells were passed through a 27G needle to obtain a single cell suspension. Splenic CD4^+^ T cells and CD19^+^ B cells from spleen and PerC were isolated from naïve mice using positive selection via magnetic-activated cell sorting using CD4- and CD19-MicroBeads (Miltenyi) respectively, and routinely between 95–99% pure based on the expression of CD4 and CD19. PerC B-1a cells were isolated by Fluorescence activated cell sorting (FACS) using a BD Influx Cell Sorter (BD Biosciences) based on the expression of CD19, CD5 and CD11b.

### Flow cytometry and intracellular cytokine staining

Single-cell suspensions were washed and subsequently stained in the presence of Fc block (2.4G2) for CD11b (M1/70), CD19 (1D3), CD4 (RM4-5), CD5 (53-7.3, biotin conjugate), CD80 (16-10A1), CD86 (GL-1), CD40 (3/23) and major histocompatibility complex (MHC) class II (M5/114.15.2) using monoclonal antibodies (mAbs) from BD or eBiosciences. After washing, the samples were analyzed on a BD FACSCanto II Flow Cytometer (BD Biosciences) and analyzed with FlowJo software (Tree Star). For intracellular cytokine staining, total cells from co-cultures were stimulated for 5 hours in the presence of 50 ng/ml phorbol 12-myristate 13-acetate (PMA), 500 ng/ml ionomycin, 1 µg/ml lipopolysaccharide (LPS; Escherichia coli 0127:B8) and 1 µg/ml Brefeldin A (BFA) (Sigma). Cells were then washed, stained extracellularly as indicated earlier, permeabilized using Cytofix/Cytoperm-solution (BD) according to manufacturer's instructions and subsequently stained intracellularly for cytokines in the presence of Fc block (2.4G2) using mAbs against IL-10 (JES5-16E3), IFN-γ (XMG1.2) and TNF-α (MP6-XT22) from BD or eBiosciences.

### *In vitro* B cell activation

B cells were *in vitro* activated using LPS (10 µg/ml), an agonistic mAb against CD40 (αCD40) (10 µg/ml; clone FGK45), a combination of these two (10 µg/ml LPS and 10 µg/ml αCD40), IL-21 (100 ng/ml recombinant protein; eBiosciences) or by α-CD40 followed by LPS (10 µg/ml) during the final 5 hours of an 48 hours culture as described in literature [9], [12], [16]. The cells were washed extensively before being used in other assays. All cultures took place in IMDM supplemented with 5×10^−5^ M 2-mercaptoethanol, penicillin (500 units/ml) and streptomycin (50 µg/ml; all from Gibco) at 37°C and 5% CO_2_ in either 96-well plates (1×10^5^/well) or 24-well plates (2×10^6^/well).

### Breg – T cell suppression assay

The suppression assay was performed as described [15], [17], briefly: indicated B cells (2×10^5^) were 1∶1 co-cultured with CD4^+^ T cells for 72 h in the presence of anti-CD3 (‘soluble’; 1 µg/ml; clone 145-2C11), while PMA, ionomycin, LPS and BFA were added during the final 5 hours of culture to facilitate intracellular cytokine staining (see above). TNF-α, IFN-γ and IL-10 positive T cells were determined intracellularly as described above.

### Luminex

Cytokines secreted during the cultures were determined by Luminex before the stimulation with PMA, ionomycin, LPS and BFA took place. Briefly, fluoresceinated microbeads coated with capture antibodies for simultaneous detection of IFN-γ (AN18), IL-2 (JES6-1A12), IL-10 (JES5-2A5), IL-17a (TC11-18H10) and IL-6 (MP5-20F3) were added to 50 µl of culture supernatant. Cytokines were detected by biotinylated antibodies against IFN-γ (XMG1.2), IL-2 (JES6-5H4), IL-10 (SXC-1), IL-17a (TC11-8H4.1) and IL-6 (MP5-32C11) followed by phycoerythrin-labeled streptavidin (All from BD Biosciences Pharmingen). Fluorescence was measured using a Luminex model 100 XYP (Luminex, Austin, TX, USA) and the cytokine concentrations were quantified using a calibration line from recombinant cytokines.

### ELISA

The presence of IgG1 and IgM in the supernatant of indicated stimulated B cells was determined by enzyme-linked immunosorbent assay (ELISA). 96-well ELISA plates (NUNC Maxisorp) were coated with polyclonal goat anti-mouse immunoglobulins (Dako Z0420). Free binding sites were blocked with a blocking buffer containing 4% horse serum (Gibco) and 1% Tween-80 (Sigma). Sera were diluted in a buffer containing 4% horse serum, 1% Tween-80 and 0.5 M NaCl, and mouse immunoglobulins were determined using horseradish peroxidase-conjugated goat anti-mouse IgG1 (Cat. 1070-05) or IgM (Cat. 1020-05, both from Southern Biotech Association).

### Statistical analysis

Statistical analysis was performed as indicated in the figure legends using GraphPad Prism (GraphPad Software Inc.).

## Results

### B-1a cells share multiple characteristics with Bregs, including the production of IL-10

Previous studies have indicated that the similarities between B-1a cells and described Bregs are not limited to their (partial) overlap in phenotype. Transgenic mice with in- or decreased amounts of certain regulatory B cell subsets often contained parallel alterations in B-1a levels, suggesting that these cells are either the same cells and/or possess similar development pathways. Similarities in reactions to certain activating triggers further supported the notion that these cells are not only phenotypically similar, but also functionally (Table S1).

**Figure 1:**
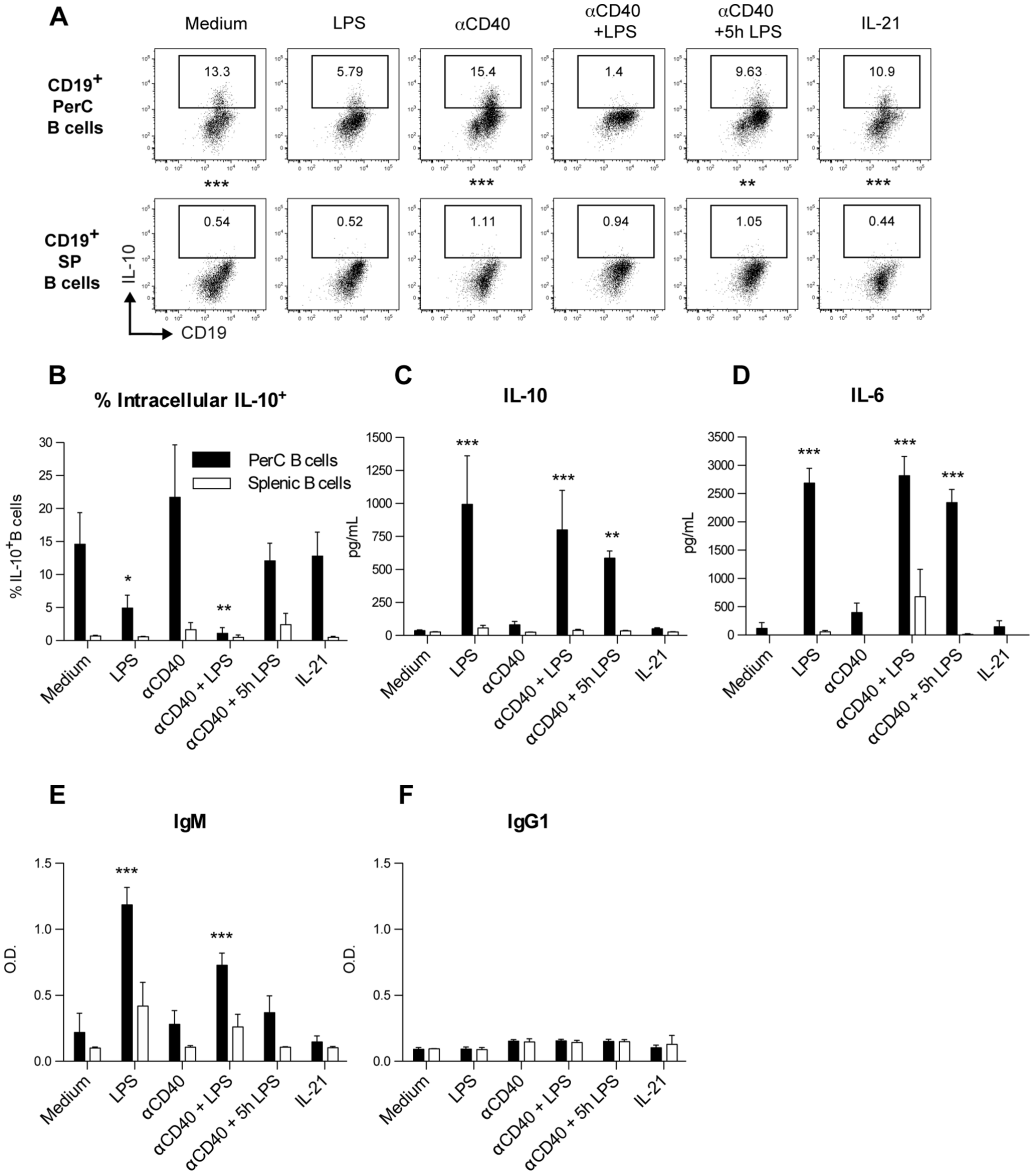
*In vitro* activated PerC B and PerC B-1a cells produce IL-10 and IL-6. Splenic B cells and PerC B cells were cultured for 48 h in the presence of medium only or indicated stimuli (see materials and methods). Intracellular IL-10 staining was performed after additional stimulation with PMA/ionomycin/LPS and both representative flow cytometric plots (A) and the percentage of IL-10^+^ B cells (B) are shown, whereas the levels of IL-10 (C), IL-6 (D), IgM (E) and IgG1 (F) were determined directly from the supernatant (before restimulation took place) using Luminex (IL-10 and IL-6) and ELISA (IgM and IgG1). Shown in B-F are the mean ± S.D. of pooled results from two identical replicate experiments (with 2–5 technical replicates each) that produced similar data. Statistically significant differences between PerC- and splenic B cells after stimulation were calculated using a two-way ANOVA with Bonferroni post-hoc test (A), whereas statistically significant difference between the various stimuli and the medium control of PerC B cells (B-F) were calculated using a one-way ANOVA with Bonferroni post-hoc test. * p <0.05, ** p<0.01, *** p<0.001.

To investigate if the B-1a cell containing PerC B cell population produced IL-10, PerC B cells and splenic B cells were stimulated for 48 h with LPS, αCD40 or a combination of these stimuli. At the end of the 48 h activation period, the cells were analyzed for their potential capacity to produce IL-10 (as determined by intracellular cytokine staining after restimulation) and the supernatant of the cells was analyzed for secreted cytokines and immunoglobulins. Figures 1A and 1B show that a significantly higher percentage of the PerC B cells possess the potential capacity to produce IL-10 after a 48 h-culture compared to Splenic B cells, either without stimulation (medium control) or after stimulation with αCD40, αCD40 in combination with a brief 5 h LPS stimulation or IL-21. None of these stimuli significantly increased the number of IL-10-producing PerC B cells compared to the medium control (Figure 1B), but treatment with LPS, αCD40+LPS or αCD40+5 hr LPS did significantly alter the produced quantities of IL-10 (Figure 1C). Analysis of IL-6 (Figure 1D) secretion into the supernatant showed that PerC B cells produced IL-6 together with IL-10. LPS and αCD40+LPS furthermore also significantly increased IgM (Figure 1E), but not IgG1 (Figure 1F) secretion by PerC B cells compared to the medium control.

### PerC B-1a cells secrete IL-10 and IL-6 similarly to the undifferentiated PerC B cell population

**Figure 2:**
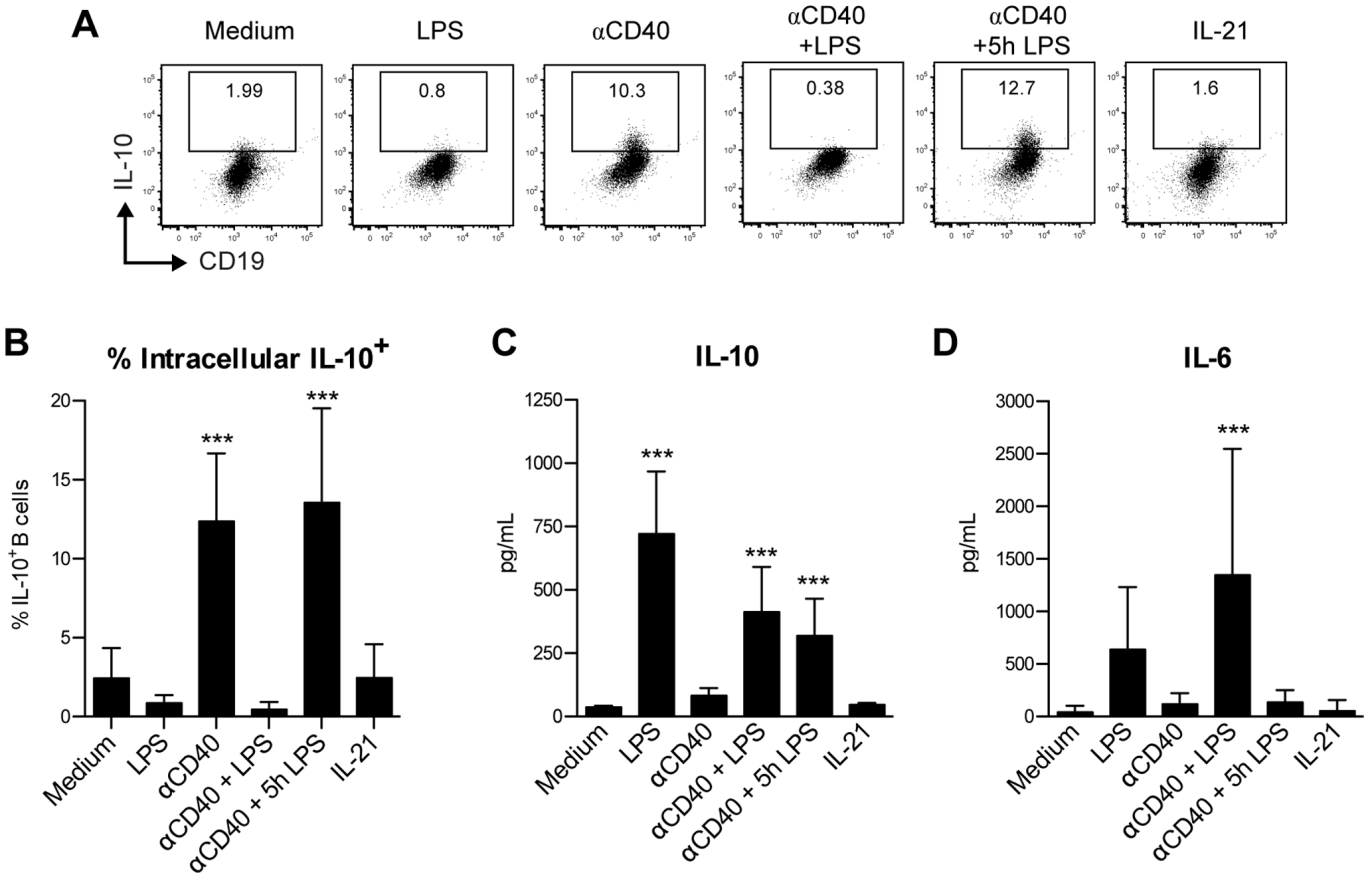
PerC B-1a cells secrete IL-10 and IL-6 similarly to the undifferentiated PerC B cell population. FACS-purified CD19^+^ CD11b^+^ CD5^+^ PerC B-1a cells were cultured for 48 h in the presence of medium or indicated stimuli. Intracellular IL-10 staining was performed after additional stimulation with PMA/ionomycin/LPS and both representative flow cytometric plots (A) and the percentage of IL-10^+^ B cells (B) are shown, whereas the levels of IL-10 (C) and IL-6 (D) were determined directly from the supernatant (before restimulation took place) using Luminex. Shown in B-D are the mean ± S.D. of pooled results from two identical replicate experiments (with 2–5 technical replicates each) that produced similar data. Statistically significant differences between the various stimuli and the medium control (B-D) of PerC B-1a cells were calculated using a one-way ANOVA with Bonferroni post-hoc test. ** p<0.01, *** p<0.001.

The PerC B cell population from BALB/c mice consist for about 65–70% out of B-1a cells [30], which suggested that the high percentage of B cells capable of IL-10-producion (Figure 1A) and the cytokines detected in the supernatant (Figure 1B-D) were at least partially derived from B-1a cells. To investigate how purified PerC B-1a cells reacted to CD40 and TLR signaling, cells were sorted prior to *in vitro* stimulation. Figure 2A and 2B indicate that the percentage of PerC B-1a cells with the potential capacity to produce IL-10 increased after a 48 h activation period with both αCD40 and αCD40+5 hr LPS. The amount of IL-10 secreted into the supernatant (Figure 2C) was significantly increased compared to the medium control after stimulation with LPS, αCD40+LPS and αCD40+5 h LPS, whereas the amount of IL-6 (Figure 2D), was only significantly different after αCD40+LPS treatment. This data supports a model in which αCD40 increases the percentage of B cells that is capable to produce IL-10, whereas LPS induces actual IL-10 secretion. This notion was described before by Lampropoulou et al. [10]. Since αCD40+5 h LPS stimulated the PerC B(-1a) cells in such as way that (i) a high percentage of the B cells possessed and maintained the capacity to produce IL-10 (Figure 1A, 1B, 2A and 2B) while (ii) high amounts of IL-10 were secreted into the supernatant (Figure 1C and 2C), the subsequent experiments were performed with this stimulation-method only.

### PerC– and splenic B cells possess different surface markers after activation

**Figure 3:**
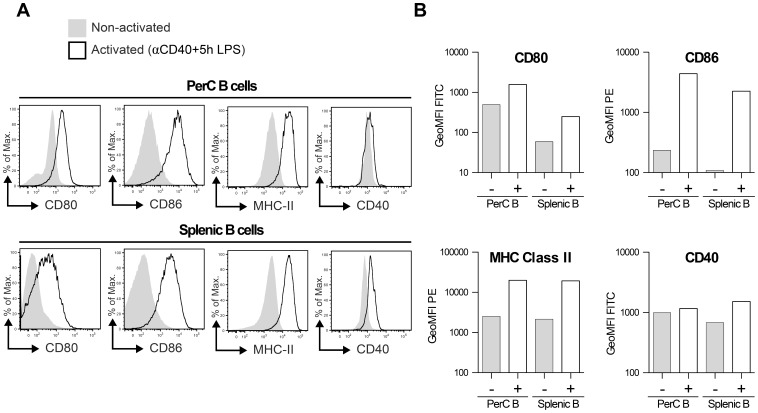
PerC– and Splenic B cells possess different surface markers after activation. Indicated B cell populations were stained extracellular with mAbs against CD19 and indicated markers after both a 48 h period of stimulation using αCD40+5 hr LPS (+: activated; see materials and methods) and after being isolated freshly (–: non-activated). Shown in A are expression levels of indicated markers on CD19^+^ B cells (representative plots of two identical replicate experiments), in B the average geometric mean fluorescence intensity (GeoMFI) levels from two identical replicate experiments.

B cells may influence T cells not solely via the secretion of cytokines but as well via direct cell-cell contact. To determine the potential differences in co-stimulative abilities between the activated and non-activated PerC- and splenic B cells, we analyzed the expression of CD80, CD86, MHC class II and CD40 of these populations. Figure 3 shows that PerC B cells express higher levels of CD80, CD86 and MHC class II compared to splenic B cells. After activation with αCD40+5 hr LPS, both subsets upregulated CD80, CD86 and MHC class II and the differences in expression between PerC B cells and splenic B cells became, although still present, much smaller. Activation upregulated CD40 expression on splenic B cells only (Figure 3).

### Activated PerC B cells suppress pro-inflammatory cytokine production by CD4^+^ T cells

**Figure 4:**
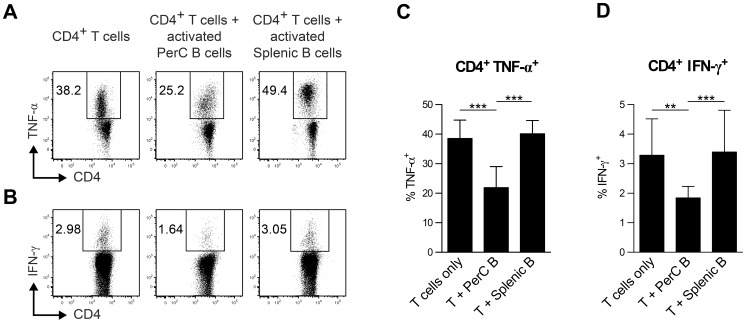
Activated PerC B cells suppress TNF-α- an IFN-γ-production by CD4^+^ T cells. Splenic- and PerC B cells were activated *in vitro* for 48 h using αCD40+5 hr LPS (see materials and methods), and co-cultured with splenic CD4^+^ T cells that were stimulated polyclonally with anti-CD3 for 72 h. The cells were additionally stimulated with PMA/ionomycin/LPS during the last 5 hours of the co-culture in order to facilitate intracellular TNF-α and IFN-γ staining. Shown are representative plots of TNF–α (A) and IFN-γ (C) production by CD4^+^ T cells, and the percentages of T cells producing TNF-α (B) and IFN-γ (D). E and F additionally show the data for indicated non-activated B cell subsets. Shown in B, D and E are the mean ± S.D. of pooled results from two identical replicate experiments (with at least triplicate technical replicates each) that produced similar data. Statistically significant differences between the various culture conditions (B and D) were calculated using a one-way ANOVA with Bonferroni post-hoc test. ** p<0.01, *** p<0.001.

Since Bregs have been described to inhibit the capacity of polyclonally activated CD4^+^ T cells to produce TNF-α and IFN-γ [15], [17] we examined the capacity of PerC B cells to do the same. The splenic B cells, which served as a control, and the PerC B cells were activated using αCD40+5 hr LPS, and subsequently added to CD4^+^ T cells that were activated with anti-CD3 mAb. The PerC B cell population was found to possess Breg functions since the added PerC B cells reduced the capacity of CD4^+^ T cells to produce both TNF-α (Figure 4A and 4B) and IFN-γ (Figure 4C and 4D), whereas splenic B cells did not show any effect.

### Activation status of B cells is critical for the effects exerted on T cells

**Figure 5:**
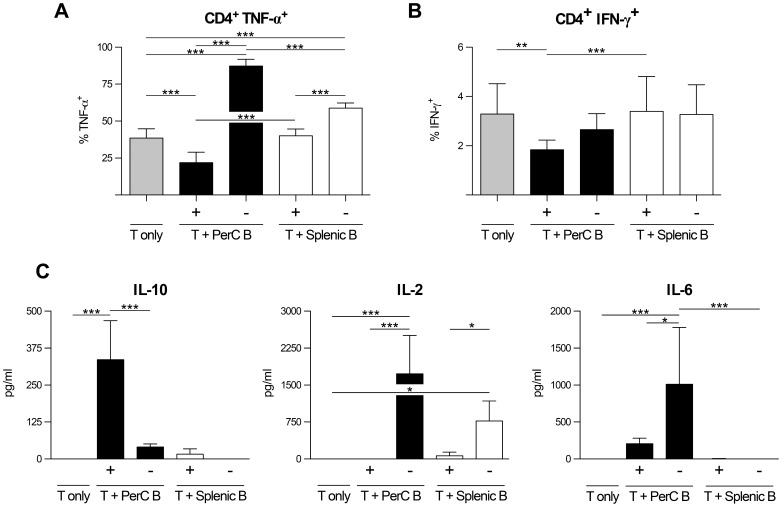
Activation status of B cells is critical for the effects exerted on T cells. B- and T cell cultures and subsequent intracellular staining were performed as explained in the legend of Figure 4. The percentages of T cells producing TNF-α (A) and IFN-γ (B) after co-culture with indicated activated (+) or non-activated (–) B cell subsets is shown. In A and B the mean ± S.D. are depicted of pooled results from two identical replicate experiments (with at least triplicate technical replicates each) that produced similar data. The cytokine levels of indicated B- and T cell cultures (C) were determined from the supernatant using Luminex. Levels shown in C are the mean ± S.D. of pooled results from two identical replicate experiments (with at least triplicate technical replicates each). Statistically significant differences between the various culture conditions were calculated using a one-way ANOVA with Bonferroni post-hoc test. *p<0.05, ** p<0.01, *** p<0.001.

Interestingly, the effect the B cells populations exerted on the activated T cells depended greatly upon the activation status of the B cells. Freshly isolated, PerC- and splenic B cells did not possess regulatory B cell roles. Instead non-activated B cells even augmented the capacity of T cells to produce TNF-α (Figure 5A) whereas the capacity to produce IFN-γ was not altered (Figure 5B).

Evaluation of the cytokine levels in the B-T cell co-cultures, indicated that co-cultures in which activated PerC B cells were used, resulted in a cytokine environment with relatively high levels of IL-10 and low levels of IL-2 and IL-6 (Figure 5C). Co-cultures in which non-activated PerC B cells were used, resulted in a cytokine environment that was characterized by the virtual absence of IL-10, but increased levels of IL-2 and IL-6 (Figure 5C). Co-cultures with non-activated splenic B cells induced IL-2 production only. No cytokines were detected when T cells were cultured alone (Figure 5C).

## Discussion

The aim of the current study was to examine the potential immunoregulatory functions of peritoneal cavity-derived B(-1a) cells. Our investigations show that peritoneal cavity-derived B cells can fulfill regulatory B cell roles *in vitro*, when activated as described [9],[12],[16]. Activation of PerC B- and B-1a cells via CD40 triggering and short term TLR4 activation induced prolonged IL-10 production and IgM secretion. Reports in the literature describe that IgM can possess regulatory functions in specific situations [31], but the complex-analysis needed to verify this possible activity is beyond the scope of the current paper.

These observations indicated that PerC B cells behave identically to functional Bregs described in the literature following a variety of stimuli [12], except for stimulation with IL-21. In our study, IL-21 did not activate PerC B(-1a) cells similarly to B10 Bregs, as this cytokine induces direct IL-10 production by the latter cells without the necessity of restimulation [9].

The co-stimulatory molecules CD80, CD86 and MHC Class II were upregulated after stimulation on both PerC- and splenic B cells, but the upregulation was greater in PerC B cells as compared to splenic B cells (Figure 3). Of note, unstimulated PerC B cells possessed significant levels of CD80, whereas splenic B cells did not. These data suggest that CD4^+^ T cells might preferentially form more and/or longer interactions with activated PerC B cells as compared to their non-activated counter parts or splenic B cells. These differences are also reflected in the powerful capabilities of innate-like B cells (e.g., B-1 cells) to serve as antigen presenting cells [32], [33].

The activation status of PerC B cells appeared to be decisive for the capacity of PerC B cells to exert regulatory B cell roles *in vitro*. Non-activated PerC B cells that were co-cultured with CD4^+^ T cells induced a predominantly pro-inflammatory environment with increased levels of B-cell derived proinflammatory IL-6 [29] and an augmented amount of TNF-α-competent T cells. Activated PerC B cells on the other hand, produce relatively more IL-10 and induced an anti-inflammatory environment with increased levels of IL-10, decreased levels of IL-6 and less TNF-α and IFN-γ-competent T cells. Future studies should point out the exact working mechanism of this PerC B cell regulatory role. Such studies may want to focus on, e.g., the IL-10-, IL-10 receptor-, dose- and contact-dependency of the described effect.

It is important to realize that the PerC B cell population contains distinct B cells; 60–70% of the PerC B cells in our BALB/c mice are B-1a cells [30]. Experiments performed with purified B-1a cells suggest that these cells are (at least partially) responsible for the IL-10 production observed when the PerC B cell subset was stimulated.

Preliminary data from experiments in which we transferred *in vitro* activated PerC- and splenic B cells to mice before the induction of proteoglycan-induced arthritis, did not demonstrate an apparent disease-dampening effect for PerC B cells whereas activated splenic B cells seemed to worsen disease (unpublished observations). CD80/86-mediated autoreactive T cell activation by B cells plays an important role in the induction of severe arthritis in this model [34]. This can explain the effect of activated splenic B cells that possess upregulated levels of CD80/86 (Figure 3). It is possible that insufficient PerC B cell numbers reached the locations necessary to suppress diseases efficiently, possibly (partly) due to interference caused by the immunization used to induce disease. However, a recent report indicates that PerC B cells can function regulatory B cell roles *in vivo* in a murine model for colitis [35].

In conventional autoimmune situations, when no cells are transferred, endogenously present PerC B cells may become stimulated *in vivo* in a similar way as splenic Bregs [4], [9]. *In vivo* stimuli for PerC B cells and splenic Bregs may include *e.g.* constitutively activated T cells that provide signals via CD40 in chronic inflammatory disease [36], a process that can take place in an antigen non-specific way [37]. The localization of B cells in the PerC does not exclude these cells from activation since PerC B cells can migrate from the PerC to the spleen [38]. Furthermore it has been suggested that PerC B cells are activated locally in the PerC after administration of distant local inflammatory mediators [39]. Difference mouse breeds might be useful model systems to analyze the contribution of PerC B-1a cells in autoimmune conditions since differences in the quantities and IL-10-producing capabilities of PerC B-1a cells between e.g. BALB/c and C57BL/6 mice have been described before [40].

B-1a cells are found in the spleen as well; splenic B-1a cells are CD11b^−^, whereas they are CD11b^+^ in the PerC, but the exact relationship between splenic- and PerC B-1a cells is not completely clear [6]. When PerC B-1a cells migrate to the spleen (e.g. after systemic stimulation with LPS), these ‘recent splenic immigrants’ are still positive for CD11b for several days [38]. Since part of the splenic B10 Bregs that are upregulated after autoimmune inflammation seem to express heightened levels of CD11b as well [5] it is tempting to speculate this population also contains activated PerC-derived B-1a cells.

PerC B-1a cells were earlier described to possess suppressive properties in a contact hypersensitivity model [41] and in a colitis model [35], [42]. We now have shown that described splenic autoimmune-suppressive Breg subsets and PerC B-1a cells might be stronger related to each other as thought before since (i) both cells reacted equally on activation-stimuli and (ii) both cells behaved similarly in a Breg suppression assay.

## Supporting Information

Table S1 **Similarities and equalities between regulatory B cells and B-1a cells.** ^1^ CD19 is a positive BCR regulator; BCR signalling is decreased in CD19^−/−^ mice, while it is vice versa increased in hCD19Tg that overexpress (human) CD19. hCD19Tg mice have increased amounts of B-1a cells, but decreased amounts of B-1b cells. CD19^−/−^ mice vice versa, possess increased amounts of B-1b cells, but lack B-1a cells [5], [6]. ^2^ CD22 is a negative BCR regulator; and it's absence results in increased BCR signaling [22]. ^3^ The regulatory B cells in these articles possess a phenotype other than the CD5^+^ CD1d^hi^ B10 cells; there are instead: MZ (CD1d^hi^) Bregs [14]/CD19^+^ IL-10^+^ CD1d^hi^ CD5^+^ CD21^hi^ CD23^+^ IgD^+^ IgM^hi^ [15]. ^4^ This article demonstrates that MZ B cells (and not FO B cells) can gain regulatory B cell roles after BAFF treatment. Splenic B-1a cells were not investigated.
(PDF)
