# Supplementary material for: Activated Peritoneal Cavity B-1a Cells Possess Regulatory B Cell Properties
Source: PLoS One. 2014 Feb 13;9(2):e88869. doi: 10.1371/journal.pone.0088869 (PMC3923827; doi:10.1371/journal.pone.0088869)
Supplement: Table S1 — Similarities and equalities between regulatory B cells and B-1a cells. 1 CD19 is a positive BCR regulator; BCR signalling is decreased in CD19−/− mice, while it is vice versa increased in hCD19Tg that overexpress (human) CD19. hCD19Tg mice have increased amounts of B-1a cells, but decreased amounts of B-1b cells. CD19−/− mice vice versa, possess increased amounts of B-1b cells, but lack B-1a cells [5], [6]. 2 CD22 is a negative BCR regulator; and it's absence results in increased BCR signaling [22]. 3 The regulatory B cells in these articles possess a phenotype other than the CD5+ CD1dhi B10 cells; there are instead: MZ (CD1dhi) Bregs [14]/CD19+ IL-10+ CD1dhi CD5+ CD21hi CD23+ IgD+ IgMhi [15]. 4 This article demonstrates that MZ B cells (and not FO B cells) can gain regulatory B cell roles after BAFF treatment. Splenic B-1a cells were not investigated. (PDF) [file pone.0088869.s001.pdf]

Table S1: Similarities and equalities between regulatory B cells and B-1a cells		
**Property**	**IL-10-producing B10 cells / Bregs with regulatory properties**	**Splenic- or PerC-derived B-1a cells**
$CD5^+$ phenotype	B10 cells are enriched within the splenic $CD5^+CD1d^{hi}$ B cell subset [1]	B-1a cells are defined by their positivity of CD5 [2]
IL-10 production after stimulation	B10 cells are functionally defined by their ability to produce IL-10 [1]	B-1 cells are known to be able to produce IL-10 [3]
$CD19^{-/-}$ mouse [1]	B10 cells are reduced by ~75% in CD19-deficient mice [1,4]	CD19-deficient lack B-1a cells [5,6]
hCD19Tg mouse [1]	B10 cells are expanded in mice that overexpress (human) CD19 [1,4]	hCD19Tg mice have an large increase in their numbers of B-1a cells [5,6]
Chronic Lymphocytic Leukemia (CLL)	CCL cells share phenotypic and functional properties with B10 cells; including their immunosuppressive function [7]	Many suggest that CCL cells are often derived from the B-1 lineage (reviewed by Nordgren et al. [8]).
$CD22^{-/-}$ mouse [2]	B10 cells are expanded in mice that lack CD22 [4,9]	B-1a cells are expanded in $CD22^{-/-}$ mice, although they seem to be functionally impaired in at least some situations [10]
CD40 ligation	B10 cells are expanded in mice with an ectopic expression of CD154 [4,9]	PerC cells vigorously proliferate *in vitro* following CD40 ligation with an agonistic antibody (unpublished observations).
1~3% of $CD19^+$ cells in spleen	About 1~3% of the splenic B cells are functionally defined B10 cells [1]	About 1~3% of the splenic B cells are B-1a cells [2]
Development dependent upon BCR diversity	B10 cells are reduced by ~90% in Tg MD4 mice with a fixed BCR specific for HEL [4]	The B-1 subset does not develop normally in MD4 mice since the PerC B cell maintain a follicular (B-2) cell phenotype [11].
Activation by the protozoa *Leishmania major*	Infection induces IL-10-producing B cells with regulatory properties and a B10 phenotype [12]	Infection causes expansion of B-1a cells *in vivo*, and B-1 cells activated *in vitro* (with leishmanial extracts) produce IL-10 [13]
Activation by the helminth *Schistosoma mansoni*	Infection induces IL-10-producing B cells [3] with regulatory properties [14] / [15]	Infection causes expansion of B-1a cell *in vivo*, and B-1 cells activated *in vitro* (with *Schistosoma mansoni* soluble egg Ag) produce IL-10 [16]
In vitro treatment with BAFF	*In vitro* treatment of splenic B cells with BAFF induces IL-10-producing regulatory B cells that suppress arthritis *in vivo* [17] [4]	*In vitro* treatment of (TLR stimulated) B-1a cells with BAFF augments IL-10 production [18]
48h *in vitro* treatment agonistic CD40 mAb and 5hr LPS	*In vitro* treatment of splenic B cells with these stimuli induces and activates IL-10-producing B10 cells to both become IL-10-competent and secrete IL-10 [4,9,19]	*In vitro* treatment of PerC B(-1a) cells with these stimuli activates these B cells in a similar way (this paper)
Co-culture of the B cells with $CD4^+$ T cells in a regulatory B cell suppression assay	Various regulatory B cells suppress TNF-α- and IFN-γ-competent $CD4^+$ T cells in a suppression assay [20,21]	PerC B(-1a) cells suppress TNF-α-and IFN-γ-competent $CD4^+$ T cells in a similar way (this paper)

[1] CD19 is a positive BCR regulator; BCR signalling is decreased in CD19$^{-/-}$ mice, while it is vice versa increased in hCD19Tg that overexpress (human) CD19. hCD19Tg mice have increased amounts of B-1a cells, but decreased amounts of B-1b cells. CD19$^{-/-}$ mice vice versa, possess increased amounts of B-1b cells, but lack B-1a cells [5,6].

[2] CD22 is a negative BCR regulator; and it's absence results in increased BCR signaling [22].

[3] The regulatory B cells in these articles possess a phenotype other than the CD5^{+} CD1d^{hi} B10 cells; there are instead: MZ (CD1d^{hi}) Bregs [14] / CD19^{+} IL-10^{+} CD1d^{hi} CD5^{+} CD21^{hi} CD23^{+} IgD^{+} IgM^{hi} [15].

[4] This article demonstrates that MZ B cells (and not FO B cells) can gain regulatory B cell roles after BAFF treatment. Splenic B-1a cells were not investigated.

References cited in Table S1

1. Yanaba K, Bouaziz JD, Haas KM, Poe JC, Fujimoto M, et al. (2008) A regulatory B cell subset with a unique CD1d^{hi}CD5^{+} phenotype controls T cell-dependent inflammatory responses. Immunity 28: 639-650.

2. Baumgarth N (2011) The double life of a B-1 cell: Self-reactivity selects for protective effector functions. Nat Rev Immunol 11: 34-46.

3. Barr TA, Brown S, Ryan G, Zhao J, Gray D (2007) TLR-mediated stimulation of APC: Distinct cytokine responses of B cells and dendritic cells. Eur J Immunol 37: 3040-3053.

4. Yanaba K, Bouaziz JD, Matsushita T, Tsubata T, Tedder TF (2009) The development and function of regulatory B cells expressing IL-10 (B10 cells) requires antigen receptor diversity and TLR signals. J Immunol 182: 7459-7472.

5. Haas KM, Poe JC, Steeber DA, Tedder TF (2005) B-1a and B-1b cells exhibit distinct developmental requirements and have unique functional roles in innate and adaptive immunity to S. pneumoniae. Immunity 23: 7-18.

6. Sato S, Ono N, Steeber DA, Pisetsky DS, Tedder TF (1996) CD19 regulates B lymphocyte signaling thresholds critical for the development of B-1 lineage cells and autoimmunity. The Journal of Immunology 157: 4371-4378.

7. DiLillo DJ, Weinberg JB, Yoshizaki A, Horikawa M, Bryant JM, et al. (2013) Chronic lymphocytic leukemia and regulatory B cells share IL-10 competence and immunosuppressive function. Leukemia 27: 170-182.

8. Nordgren T, Joshi S (2010) The etiology of chronic lymphocytic leukemia: Another look at the relationship between B1 cells and CLL. The Open Leukemia Journal 3: 69-73.

9. Poe JC, Smith SH, Haas KM, Yanaba K, Tsubata T, et al. (2011) Amplified B lymphocyte CD40 signaling drives regulatory B10 cell expansion in mice. PLoS One 6: e22464.

10. Nakashima H, Hamaguchi Y, Watanabe R, Ishiura N, Kuwano Y, et al. (2010) CD22 expression mediates the regulatory functions of peritoneal B-1a cells during the remission phase of contact hypersensitivity reactions. J Immunol 184: 4637-4645.

11. Miles K, Heaney J, Sibinska Z, Salter D, Savill J, et al. (2012) A tolerogenic role for toll-like receptor 9 is revealed by B-cell interaction with DNA complexes expressed on apoptotic cells. Proceedings of the National Academy of Sciences 109: 887-892.

12. Ronet C, Torre YH, Revaz-Breton M, Mastelic B, Tacchini-Cottier F, et al. (2010) Regulatory B cells shape the development of Th2 immune responses in BALB/c mice infected with leishmania major through IL-10 production. The Journal of Immunology 184: 886-894.

13. Palanivel V, Posey C, Horauf AM, Solbach W, Piessens WF, et al. (1996) B-cell outgrowth and ligand-specific production of IL-10 correlate with Th2 dominance in certain parasitic diseases. Exp Parasitol 84:

168-177.

14. van dV, Labuda LA, Ozir-Fazalalikhan A, Lievers E, Gloudemans AK, et al. (2012) Schistosomes induce regulatory features in human and mouse $CD1d^{hi}$ B cells: Inhibition of allergic inflammation by IL-10 and regulatory T cells. PLoS ONE 7: e30883.

15. Amu S, Saunders SP, Kronenberg M, Mangan NE, Atzberger A, et al. (2010) Regulatory B cells prevent and reverse allergic airway inflammation via FoxP3-positive T regulatory cells in a murine model. J Allergy Clin Immunol 125: 1114-1124.e8.

16. Velupillai P, Secor WE, Horauf AM, Harn DA (1997) B-1 cell ($CD5^{+}B220^{+}$) outgrowth in murine schistosomiasis is genetically restricted and is largely due to activation by polylactosamine sugars. J Immunol 158: 338-344.

17. Yang M, Sun L, Wang S, Ko K, Xu H, et al. (2010) Cutting edge: Novel function of B cell-activating factor in the induction of IL-10–Producing regulatory B cells. The Journal of Immunology 184: 3321- 3325.

18. Ng L, Ng C, Woehl B, Sutherland AP, Huo J, et al. (2006) BAFF costimulation of toll-like receptor-activated B-1 cells. Eur J Immunol 36: 1837-1846.

19. Yoshizaki A, Miyagaki T, DiLillo DJ, Matsushita T, Horikawa M, et al. (2012) Regulatory B cells control T-cell autoimmunity through IL-21-dependent cognate interactions. Nature 491: 264-268.

20. Blair PA, Chavez-Rueda KA, Evans JG, Shlomchik MJ, Eddaoudi A, et al. (2009) Selective targeting of B cells with agonistic anti-CD40 is an efficacious strategy for the generation of induced regulatory T2-like B cells and for the suppression of lupus in MRL/lpr mice. J Immunol 182: 3492-3502.

21. Matsushita T, Horikawa M, Iwata Y, Tedder TF (2010) Regulatory B cells (B10 cells) and regulatory T cells have independent roles in controlling experimental autoimmune encephalomyelitis initiation and late-phase immunopathogenesis. J Immunol 185: 2240-2252.

22. Kalampokis I, Yoshizaki A, Tedder TF (2013) IL-10-producing regulatory B cells (B10 cells) in autoimmune disease. Arthritis Res Ther 15 Suppl 1: S1.
